# A Case of Inversion of the Uterus

**Published:** 1861-09

**Authors:** Henry Nichell


ART. III.—*A Case of Inversion of the Uterus, by Henry Nichell, M. D.*

Having read several articles about reduction of inverted uterus, at different periods, varying from a few minutes to many years standing I desire to communicate through the medium of your valuable Journal, the brief history of a case which came under my observation and care in 1846, in private practice, in the city of Mainz, in Germany, while I was yet a young practitioner. At the time I was not fully impressed with its importance, but acted as I thought proper, feeling that this state of things should be remedied in some way.

I was called one afternoon to a middle aged woman, a widow of small stature, apparent good health and spirits, who told me she had a swelling in her private parts, of many years standing, which had only troubled her for a few days recently, though when it first made its appearance, some fifteen years since, it caused great pain, soreness, discharge and much bleeding, making her very unwell. That it came gradually upon her immediately after her last confinement ; that all these difficulties gradually subsided, and she finally became quite well, the tumor still remaining.

On examination I was surprised to find a tumor nearly as large as a small infant's head at birth, protruding through the vulva, and resting between the thighs. Its surface presented the appearance of common thick integument, upon the posterior wall there was some slight excoriation arising from the friction to which it had been subjected; upon pressure no pain was experienced, her only complaint was, that in walking, she felt pain, especially in the lower part of the abdomen and groins, with frequent desire to void urine.

At first I was inclined to consider it a large polypus, but upon careful examination per vagunum et rectum, was fully satisfied that it was an inverted uterus. Supposing the tumor could not be reduced on account of its great size. I ordered application of aqua plumbi to be constantly made to the excoriation, and the whole tumor, which had the pleasant effect of corrugating the surface, and greatly reducing the size of the organ, so that after the lapse of three days, I was able to restore the inverted uterus to its proper position; I grasped the tumor and compressing it gradually with firm upward pressure, carried it into the vaginal canal, I then used the thumb and two fingers pressing it onwards, until at last the womb seemed to be in its natural position.

So far as I recollect, I succeeded without very much trouble in accomplishing this result. I then introduced a round pessary, and kept the woman at rest for some days. I have seen her several times since, and always found her enjoying excellent health.

My recollection of this case has greatly increased my interest in the cases which have been reported in the journals for the last few years by Drs. West, Tyler Smith, Bockendahl, and James P. White, of this city, whose reports have been exceedingly interesting and instructive.

